# Association of CONUT Score with Ultrasound-Measured Muscle Mass and 30-, 45-, and 60-Day Mortality in Geriatric Palliative Care Patients: A Prospective Observational Study

**DOI:** 10.3390/jcm15010171

**Published:** 2025-12-25

**Authors:** Ayfer Durak, Umut Safer

**Affiliations:** 1 Department of İnternal Medicine, Division of Geriatrics, Sancaktepe Şehit Prof. Dr. Ilhan Varank Training and Research Hospital, 34785 Istanbul, Türkiye; umutsafer@gmail.com; 2 Department of İnternal Medicine, Division of Geriatrics, Amasya University Sabuncuoğlu Şerefeddin Training and Research Hospital, 05100 Amasya, Türkiye

**Keywords:** muscle wasting, malnutrition, palliative care, ultrasonography, CONUT score, muscle mass, mortality

## Abstract

**Background/Objectives**: Early risk assessment of nutritional and muscular status in geriatric palliative care patients may facilitate timely, personalized care. This study aimed to evaluate the association between the CONUT score, ultrasound-assessed muscle mass, and short-term mortality at 30, 45, and 60 days. **Methods**: This prospective, single-center study was conducted in a tertiary palliative care unit between May and September 2024. Muscle mass was assessed via ultrasound by measuring the thickness and cross-sectional area of the rectus femoris and biceps brachii. Nutritional status was evaluated using the CONUT score (low: ≤4, high: ≥5). Mortality at 30, 45, and 60 days was analyzed using Kaplan–Meier curves, Cox regression, and ROC analysis. **Results**: In a cohort of 200 geriatric palliative care patients (mean age 78.4 ± 10.2 years; 54.5% female), those with higher CONUT scores (≥5) had significantly lower ultrasound-assessed muscle mass and higher short-term mortality rates (48.6% vs. 11.3%, *p* < 0.001). A CONUT score ≥ 5 and the presence of malignancy independently predicted mortality, with hazard ratios up to 3.72. The CONUT score demonstrated moderate predictive accuracy for short-term mortality, highest at 60 days (AUC = 0.736). Kaplan–Meier analysis revealed significantly reduced survival among patients with higher CONUT scores. **Conclusions**: A CONUT score ≥ 5 and malignancy independently predicted short-term mortality, and higher CONUT scores were associated with lower muscle mass. The CONUT score may be a useful tool for early risk assessment in geriatric palliative care, though further research is warranted.

## 1. Introduction

The increasing prevalence of chronic diseases, population aging, and the expansion of treatment options have led to a growing demand for palliative care services [1].

In this context, advanced age, multiple comorbidities, and disease-related metabolic alterations are among the main risk factors for the development of malnutrition and muscle loss in palliative care patients [2].

These conditions are closely associated with adverse clinical outcomes, including increased susceptibility to infections, falls, reduced functional capacity, and higher mortality risk [3,4]. Although malnutrition and muscle loss frequently coexist and share similar adverse outcomes, they represent distinct clinical entities. Malnutrition primarily reflects inadequate nutrient intake or absorption [3], whereas muscle loss—often related to sarcopenia or cachexia—may occur even in the presence of adequate nutrition due to inflammation, aging, and other catabolic processes [4]. In palliative care patients, differentiating between these conditions is essential for appropriate intervention planning and prognostication [3,4].

In these patients with limited life expectancy, early assessment of nutritional status and muscle mass [2] may contribute to maintaining quality of life by facilitating timely nutritional support, personalized physical activity recommendations, and tailored care strategies [5].

Indeed, a recent systematic review and meta-analysis by Chan-Fook et al. (2025) demonstrated that the early initiation of therapeutic exercise programs in hospitalized sarcopenic adults significantly improves functional capacity and muscle strength [6].

Similarly, in a prospective cohort study, Rinninella et al. reported that nutritional support initiated within 48 h of hospital admission was significantly associated with a reduced risk of in-hospital mortality [7]. Although these findings are based on non-palliative populations, similar approaches may be beneficial in palliative care when carefully adapted to individual needs and goals.

Moreover, early prognostic assessment may enhance prognostic awareness among patients and their families, facilitating shared decision-making and collaborative care planning with caregivers [8] thereby promoting holistic care and more efficient use of healthcare resources [1].

However, in the palliative care setting, patient-specific clinical complexities and the limited feasibility of conventional assessment tools can hinder the timely and practical evaluation of nutritional status and muscle mass. In particular, physical frailty, functional limitations, and multiple comorbidities may restrict the use of standard approaches such as anthropometric measurements, detailed dietary assessments, and advanced imaging methods [9].

Thus, there is a need for assessment tools that are practical, easy to implement at the bedside, and feasible for use in frail populations. In this context, the CONUT score and muscle ultrasonography have emerged as promising options due to their ease of use and capacity to yield quantifiable assessments of nutritional and muscular status [10,11].

The Controlling Nutritional Status (CONUT) score is a screening tool that evaluates nutritional status based on routine laboratory parameters, including serum albumin, total lymphocyte count, and total cholesterol. It provides a multidimensional assessment of nutritional and immunological status by indirectly reflecting protein reserves (albumin), caloric and lipid metabolism (cholesterol), and immune competence (lymphocyte count) [10].

High CONUT scores have been associated with reduced skeletal muscle mass and elevated systemic inflammatory response [12]. Several nutritional screening and prognostic tools, including the Mini Nutritional Assessment–Short Form (MNA-SF), Nutritional Risk Screening 2002 (NRS-2002), Malnutrition Universal Screening Tool (MUST), and Geriatric Nutritional Risk Index (GNRI), are widely used in older adults. However, these instruments often rely on subjective assessments, functional status, or anthropometric measurements, which may be challenging to obtain or less reliable in palliative care settings. In this context, the CONUT score, being entirely based on objective laboratory parameters, may represent a practical and reproducible option for nutritional and prognostic assessment in clinically unstable older patients, including those commonly encountered in palliative care settings [10,12,13].

Furthermore, studies across diverse patient populations—including those with stroke [14], head and neck cancer [15], or undergoing dialysis [16]—have consistently shown that elevated CONUT scores are linked to higher morbidity and mortality.

However, research specifically examining the association between the CONUT score, muscle mass, and short-term mortality in geriatric palliative care populations remains limited.

In assessing muscle mass, ultrasonography has emerged as a cost-effective and clinically feasible alternative to advanced imaging techniques such as computed tomography (CT), magnetic resonance imaging (MRI), and dual-energy X-ray absorptiometry (DXA) [11]. Given its portability, bedside applicability, and safety, ultrasonography is increasingly recognized as a practical tool for muscle assessment in frail patients [11], and may be particularly suitable for use in palliative care settings.

Measurements of muscle thickness or cross-sectional area—especially in superficial muscles such as the quadriceps femoris and biceps brachii—have been shown to correlate significantly with functional capacity, nutritional status, and short-term prognosis [11].

Additionally, in hospitalized older adults, nutritional and inflammatory biomarkers are important indicators of muscle health and functional status [12]. Therefore, assessing both muscle mass and nutritional status may offer a more comprehensive understanding of clinical risk, particularly in frail populations [17].

This study aims to evaluate the relationship between the CONUT score, ultrasound-assessed muscle mass, and short-term mortality at 30, 45, and 60 days. The findings may provide preliminary insights to support early risk identification and inform clinical decision-making in geriatric palliative care.

## 2. Materials and Methods

### 2.1. Study Design and Ethical Committee Approval

This was a single-center, prospective, observational cohort study conducted between May and September 2024 in a tertiary-level palliative care unit. The study adhered to the principles of the Declaration of Helsinki and was approved by the local institutional ethics committee (Approval No: 2024/134). No financial incentives were offered to participants.

### 2.2. Participants and Eligibility Criteria

Geriatric palliative care patients who were consecutively admitted were screened for eligibility. Written informed consent was obtained from each patient or their legal representative. Patients who were unable or unwilling to provide consent were excluded. Additional exclusion criteria included

- Ongoing chemotherapy;
- Acute infection;
- Use of cholesterol-lowering medications;
- Chronic renal failure;
- Liver cirrhosis;
- Any condition that could impair ultrasonographic measurement accuracy, such as right-sided hemiplegia, contracture, edema, or fracture.

**Figure 1:**
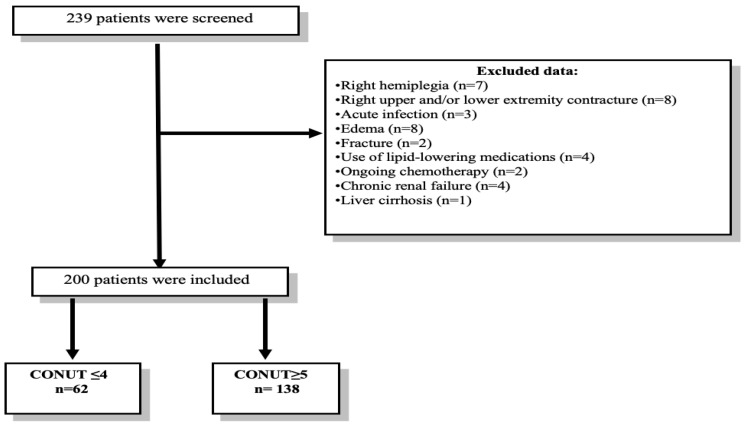
A flow diagram of the patient selection procedure.

To standardize the measurements, all ultrasound assessments were conducted on the right side of the body (Figure 1).

### 2.3. Sample Size Calculation

Sample size was calculated using the G*Power software (version 3.1), based on previously reported mortality rates according to CONUT scores [18]. Assuming a medium effect size (Cohen’s w = 0.30), 80% statistical power, and a two-sided alpha of 0.05, the minimum required sample was 80 participants. To account for a possible 10% attrition rate, 239 patients were enrolled. Following exclusions, 200 patients remained for final analysis.

### 2.4. Study Variables and Measurements

#### 2.4.1. Demographic and Clinical Variables

The following data were collected at baseline: age, sex, body mass index (BMI), nutritional route (Oral Vs. Enteral/Parenteral), comorbidities including hypertension, diabetes, malignancy, stroke and dementia; Charlson Comorbidity Index (CCI); total survival time; and mortality status at 30, 45, and 60 days. Functional status was assessed using the Katz Index of Activities of Daily Living (ADL), which scores six basic activities (bathing, dressing, toileting, transferring, continence, and feeding). Each is scored as 0 (dependent) or 1 (independent), yielding a total score from 0 to 6. Scores < 6 indicate dependency [19].

The body weights of ambulatory patients were measured using a digital scale (TANITA BC-418, Tanita Corp., Tokyo, Japan), and their heights were measured without shoes using a portable stadiometer (Seca 213, Seca GmbH, Hamburg, Germany). For bedridden patients, body weight was measured using a hoist scale (Seca Model 985, Seca GmbH, Hamburg, Germany), and height was measured in the supine or lateral position using a non-flexible measuring tape (Seca 201, Seca GmbH, Hamburg, Germany). BMI was calculated as weight (kg) divided by height squared (m^2^). According to ESPEN criteria, low BMI was defined as <20 kg/m^2^ for patients < 70 years and <22 kg/m^2^ for those ≥ 70 years [20].

#### 2.4.2. Laboratory Assessments

All blood samples were obtained via venous sampling in the morning, at the time of admission to the palliative care unit, after at least 8 h of fasting. Hemoglobin (g/dL) and lymphocyte (×10^9^/L) levels, as part of the complete blood count parameters, were measured using the Mindray BC-6800 hematology analyzer (Mindray Bio-Medical Electronics Co., Ltd., Shenzhen, China). Serum biochemistry parameters including albumin [g/L, colorimetric method], C-reactive protein (CRP) [mg/L, immunoturbidimetric method], total cholesterol (mg/dL), and creatinine (mg/dL) levels were analyzed using the Roche Cobas 6000 modular analyzer system (Roche Diagnostics GmbH, Mannheim, Germany).

#### 2.4.3. Ultrasound Muscle Assessment

Muscle ultrasonography was performed by a single experienced physician, who was blinded to clinical data. Measurements were obtained within the first 24 h of admission in patients who had achieved clinical stabilization, or as soon as stabilization was reached in those requiring a longer period. To enhance consistency and reproducibility, assessments were not conducted during periods of acute clinical instability, such as hemodynamic compromise or respiratory distress.

B-mode imaging was conducted with a linear probe (4–12 MHz; 5 cm width; Philips Affiniti 50, Philips Healthcare) (Amsterdam, The Netherlands), following guidelines from the European Geriatric Medicine Society Sarcopenia Special Interest Group [21].

During the measurements, patients were evaluated in the supine position with their extremities in full extension, following a 5 min rest period to minimize muscle activity. Measurements of muscle and subcutaneous fat tissue were performed on the rectus femoris at the distal third point between the anterior inferior iliac spine and the superior border of the patella [22] and on the biceps brachii at the midpoint between the acromion and the olecranon, using minimal probe pressure [23].

**Figure 2:**
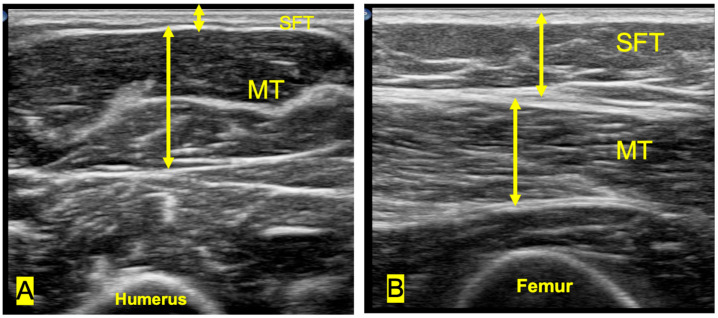
(**A**) Transverse ultrasonographic image of the biceps brachii muscle. (**B**) Transverse ultrasonographic image of the rectus femoris muscle. MT, muscle thickness; SFT, subcutaneous fat thickness.

For both muscle groups, three parameters were measured: subcutaneous fat thickness (SFT), muscle thickness (MT), and cross-sectional area (CSA). SFT was defined as the distance between the skin and the superficial fascia (cm); MT as the distance between the superficial and deep fascia (cm); and CSA as the muscle area enclosed between these two fascial layers (cm^2^) (Figure 2).

Each parameter was measured three times per patient, and the average was used in analyses. In a separate healthy subgroup (*n* = 15), intra-rater reliability was assessed with repeated measurements after 15 min. Intraclass correlation coefficients (ICC) were as follows: RF-MT = 0.95, RF-CSA = 0.93, BB-MT = 0.90, BB-CSA = 0.95—indicating high reliability.

#### 2.4.4. CONUT Score Calculations and Grouping

The CONUT score is a nutritional screening tool developed by Ulíbarri et al. [10] based on serum albumin concentration, total lymphocyte count, and total cholesterol level, yielding a total score ranging from 0 to 12. According to established thresholds, a score of 0–1 reflects normal nutritional status, 2–4 mild malnutrition, 5–8 moderate malnutrition, and 9–12 severe malnutrition (Table 1).

Nutritional status was assessed at the time of admission using the CONUT score.

Patients were grouped as

- Low CONUT: score ≤ 4 (normal–mild malnutrition);
- High CONUT: score ≥ 5 (moderate–severe malnutrition).

The grouping of CONUT scores (≤4 vs. ≥5) was based on prior studies and meta-analyses that have commonly classified scores ≥ 5 as indicative of moderate-to-severe malnutrition [14].

All participants were prospectively followed for all-cause mortality at 30, 45, and 60 days. These predefined time points were selected in light of their practical utility in geriatric palliative care, where short-term survival expectations often inform important clinical decisions.

### 2.5. Statistical Analysis

Statistical analyses were performed using IBM SPSS Statistics software (version 27.0; IBM Corp., Armonk, NY, USA). The distribution of continuous variables was assessed using the Kolmogorov–Smirnov test. Variables with a normal distribution were presented as mean ± standard deviation, while those without a normal distribution were presented as median (interquartile range, IQR). Categorical variables were expressed as frequencies and percentages (%). For comparisons between groups, the independent samples *t*-test was used for normally distributed data, while the Mann–Whitney U test was applied for non-normally distributed data. Pearson’s chi-square test was used for the analysis of categorical variables. The relationships between the CONUT score and muscle parameters (RF-MT, RF-CSA, BB-MT, BB-CSA) were examined using Spearman correlation analysis according to the distribution characteristics of the variables

Univariate and multivariable Cox regression analyses were performed to identify inde pendent risk factors associated with mortality. Variables identified as statistically significant but showing high collinearity were not included in the multivariable model. Multicollinearity was assessed using the variance inflation factor (VIF), and no significant multicollinearity was detected among the included variables (all VIF values < 2.0). In addition, Pearson correlation analyses were performed to examine collinearity between related ultrasound parameters. Strong correlations were observed between RF-MT and RF-CSA (r = 0.830, *p* < 0.001), and between BB-MT and BB-CSA (r = 0.829, *p* < 0.001). To avoid redundancy, only MT (muscle thickness) values were included in the multivariable model. The selection of variables for the multivariable Cox regression model was guided by a combination of statistical significance in univariate analysis and observed baseline differences across CONUT risk categories (Table 2). Age was retained due to its established prognostic relevance in older adults, despite not reaching statistical significance. Functional dependency was excluded to avoid multicollinearity, given its clinical and statistical overlap with comorbidity and malignancy. The individual components of the CONUT score (serum albumin, total cholesterol, and lymphocyte count) were not included separately in the multivariable model to avoid conceptual overlap. The final model included age, sex, BMI, hemoglobin, CCI score, presence of cancer, CONUT score ≥ 5, RF-SFT, RF-MT, and BB-MT. The findings were presented as hazard ratios (HR) with 95% confidence intervals (CI).

The predictive ability of the CONUT score for 30-, 45-, and 60-day mortality was evaluated using receiver operating characteristic (ROC) analysis. Diagnostic accuracy was assessed using the area under the curve (AUC). The AUC values were 0.694 (95% CI: 0.614–0.773; *p* < 0.001) for 30 days, 0.706 (95% CI: 0.633–0.779; *p* = 0.001) for 45 days, and 0.736 (95% CI: 0.667–0.804; *p* = 0.001) for 60 days. Survival analyses were performed using the Kaplan–Meier method, and differences between groups were evaluated with the log-rank test. Mean and median survival times were reported with 95% confidence intervals. Mean survival times were calculated across the entire follow-up duration and are reported as descriptive indicators of overall survival. These values are not intended to replace the predefined 30-, 45-, and 60-day mortality endpoints, but rather to complement them by providing a broader perspective. There were no missing data in the variables included in the statistical analyses; therefore, no imputation or exclusion for missingness was required. A *p*-value < 0.05 was considered statistically significant in all analyses.

## 3. Results

### 3.1. Baseline Characteristics of Patients and Between-Group Comparisons

The mean age of the 200 patients included in the study was 78.4 ± 10.2 years (range: 55–100), and 54.5% of the participants were female. Significant differences were observed between the two groups formed according to the CONUT score in terms of demographic, clinical, and biochemical characteristics. In patients with a high CONUT score, male gender (*p* = 0.027), functional dependency as assessed by ADL (*p* < 0.001), and the presence of malignancy (*p* < 0.001) were more frequently observed. Notably, baseline characteristics such as malignancy and functional dependency showed significant differences between CONUT groups, which may indicate potential confounding and should be considered when interpreting group-level comparisons.

Mortality rates also differed significantly according to the CONUT score. Among patients with a high CONUT score, 30-, 45-, and 60-day mortality rates were 34.1%, 42.0%, and 48.6%, respectively. In contrast, mortality rates in the low CONUT score group ranged between 9.7% and 11.3%. Intergroup differences were statistically significant at all assessed time points (*p* < 0.001).

Analysis of biochemical parameters revealed that serum albumin, total cholesterol, lymphocyte count, and hemoglobin levels were significantly lower in the high CONUT score group (*p* < 0.001 for all comparisons). Conversely, C-reactive protein (CRP) levels were found to be significantly elevated in this group (*p* < 0.001).

In terms of muscle measurements, patients with a high CONUT score had significantly lower values for rectus femoris subcutaneous fat thickness (RF-SFT; *p* = 0.008), rectus femoris muscle thickness (RF-MT; *p* < 0.001), rectus femoris cross-sectional area (RF-CSA; *p* < 0.001), biceps brachii muscle thickness (BB-MT; *p* = 0.006), and biceps brachii cross-sectional area (BB-CSA; *p* = 0.013) (Table 2).

### 3.2. Correlation Between Muscle Parameters and CONUT Score

According to Spearman correlation analysis, weak to moderate statistically significant negative correlations were observed between CONUT scores and muscle parameters. The strongest negative correlations were found with rectus femoris cross-sectional area (RF-CSA; r = –0.308, *p* < 0.001) and rectus femoris muscle thickness (RF-MT; r = –0.271, *p* = 0.001). Similarly, significant negative correlations were also found with biceps brachii muscle thickness (BB-MT; r = –0.261, *p* < 0.001) and biceps brachii cross-sectional area (BB-CSA; r = –0.240, *p* = 0.001) (Table 3).

### 3.3. Cox Regression Analysis

Univariate Cox regression analysis revealed that CCI score, presence of malignancy, and a CONUT score ≥ 5 were significantly associated with mortality. However, in the multivariable analysis, only malignancy and a CONUT score ≥ 5 remained independent predictors of mortality. For 30-day mortality, a CONUT score ≥ 5 was associated with a significantly increased risk of death (HR = 2.862; 95% CI: 1.212–6.755; *p* = 0.016). Similarly, the presence of malignancy was also identified as a significant risk factor (HR = 3.025; 95% CI: 1.741–5.256; *p* < 0.001). In the 45-day mortality analysis, both CONUT ≥ 5 (HR = 3.043; 95% CI, 1.380–6.711; *p* < 0.001) and malignancy (HR = 3.132; 95% CI, 1.903–5.155; *p* = 0.006) remained statistically significant predictors. In the 60-day analysis, CONUT ≥ 5 (HR = 3.723; 95% CI, 1.702–8.143; *p* < 0.001) and the presence of malignancy (HR = 2.826; 95% CI, 1.777–4.494; *p* < 0.001) continued to show significant associations with mortality (Table 4).

### 3.4. ROC Curve Analysis

**Figure 3:**
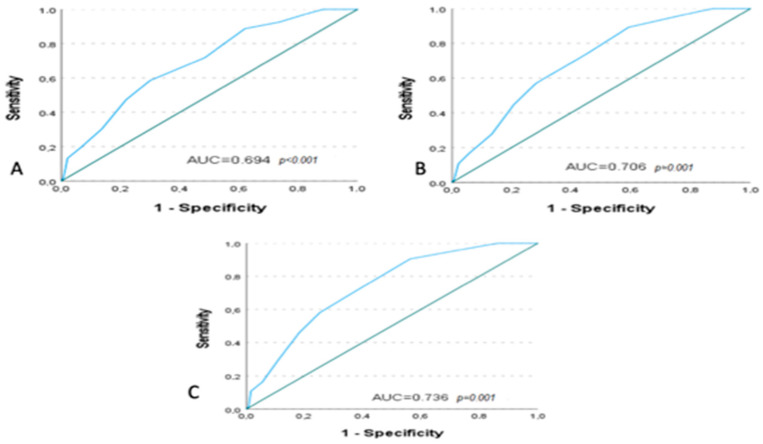
Receiver operating characteristics (ROC) curves for predicting outcome based on CONUT score. (**A**) 30-day outcome (AUC = 0.694, 95% CI: 0.614–0.773, *p* < 0.001). (**B**) 45-day outcome (AUC = 0.706, 95% CI: 0.633–0.779, *p* = 0.001). (**C**) 60-day outcome (AUC = 0.736, 95% CI: 0.667–0.804, *p* = 0.001). AUC: Area Under the Curve.

Receiver operating characteristic (ROC) analysis was performed to evaluate the predictive performance of the CONUT score for short-term mortality outcomes. The CONUT score demonstrated acceptable discriminatory ability for 30-day mortality with an AUC of 0.694 (95% CI: 0.614–0.773; *p* < 0.001). For 45-day mortality, the AUC was 0.706 (95% CI: 0.633–0.779; *p* = 0.001). The highest predictive performance was observed for 60-day mortality, with an AUC of 0.736 (95% CI: 0.667–0.804; *p* = 0.001). These results indicate that the CONUT score has a moderate but clinically relevant ability to predict short-term mortality, particularly over a 60-day follow-up period (Figure 3).

To improve the clinical applicability of the ROC analysis, we additionally reported the optimal cut-off values, sensitivity, specificity, Youden Index, and predictive values of the CONUT score. For 30-day mortality, the optimal cut-off was 6.50 (sensitivity: 58.5%, specificity: 70.1%, PPV: 41.3%, NPV: 82.4). For 45-day and 60-day mortality, a lower cut-off of 4.50 yielded higher sensitivity (89.2% and 90.5%, respectively), but lower specificity (40.7% and 43.7%, respectively). The highest Youden Index (0.342) and PPV (48.6%) were observed for 60-day mortality, further supporting its clinical usefulness in longer follow-up periods (Table 5).

### 3.5. Kaplan–Meier Survival Analysis by CONUT Score

**Figure 4:**
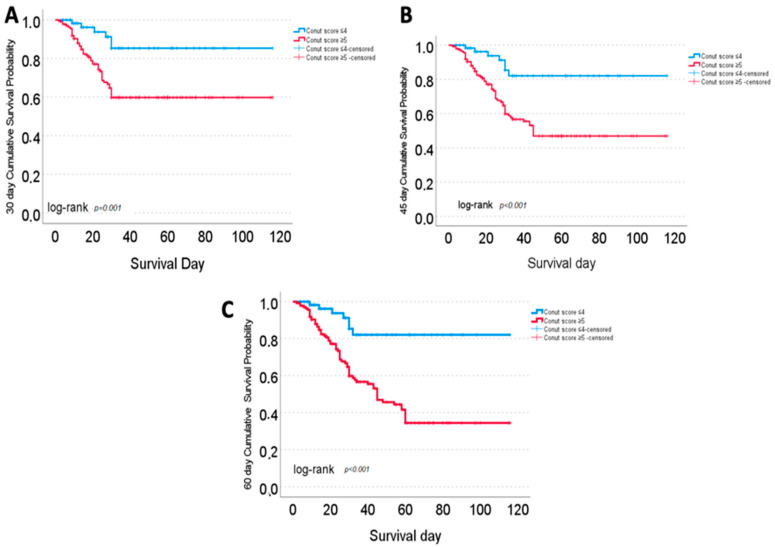
Kaplan–Meier survival curves showing 30-day (**A**), 45-day (**B**), and 60-day (**C**) mortality rates according to CONUT score groups (≤4 and ≥5). Survival rates were significantly lower in the CONUT ≥ 5 group at all three time points (log-rank test, *p* < 0.001).

Kaplan–Meier analysis was conducted to evaluate the impact of the CONUT score on survival over 30-, 45-, and 60-day follow-up periods. Individuals with a high CONUT score (≥5) exhibited significantly lower survival rates at all three time points (log-rank *p* ≤ 0.001). In the 30-day analysis, the survival rate was 85.4% in the low CONUT group and 59.8% in the high CONUT group. The mean survival times were 102.4 days (95% CI, 92.3–112.6) and 97.1 days (95% CI, 85.2–109.0), respectively; the median survival time was not reached in either group. At 45 days, survival remained at 82.1% in the low CONUT group, while it declined to 46.9% in the high CONUT group. The corresponding mean survival times were 99.7 days (95% CI, 88.5–110.8) and 83.1 days (95% CI, 70.9–95.3), respectively. At this time point, the median survival time for the high CONUT group was identified as 45 days. By day 60, survival in the low CONUT group remained stable at 82.1%, whereas it further decreased to 34.5% in the high CONUT group. The mean survival times were 99.7 days (95% CI, 88.5–110.8) for the low CONUT group and 71.6 days (95% CI, 59.8–83.4) for the high CONUT group. The median survival time in the high CONUT group remained at 45 days (Figure 4).

## 4. Discussion

### 4.1. Main Study Outcomes

This prospective study investigated the relationship between nutritional status, muscle mass, and short-term mortality in geriatric palliative care patients using two simple, bedside-applicable tools: the Controlling Nutritional Status (CONUT) score and ultrasound-based muscle assessment. The results demonstrated that higher CONUT scores were associated with reduced muscle mass, while both a high CONUT score (≥5) and the presence of malignancy independently predicted 30-, 45-, and 60-day mortality.

These findings suggest that the CONUT score may indicate underlying nutritional and inflammatory conditions, potentially explaining its prognostic value in geriatric palliative patients. Given its ease of use, objectivity, and routine laboratory basis, the CONUT score may be a useful marker for identifying patients at risk stratification and care planning in palliative care settings.

### 4.2. Malnutrition Prevalence and Clinical Correlates by CONUT Score

According to the CONUT scoring system, approximately 69% of the patients in our cohort were classified as having moderate to severe malnutrition (CONUT ≥ 5). The high prevalence of malnutrition may be associated with age-related physiological changes (e.g., sensory decline, gastrointestinal symptoms, cognitive impairment), as well as disease-specific factors (e.g., chronic inflammation, organ failure) [3]. This aligns with previously reported rates in hospitalized older adults, such as the 61.5% prevalence described by Lo Buglio et al. [12]. These findings may indicate the importance of early nutritional screening and intervention, particularly in older adults with multiple comorbidities.

Our findings suggest that higher CONUT scores may be associated with increased clinical and biological indicators of vulnerability in geriatric palliative care patients. Rather than merely reflecting isolated laboratory abnormalities, elevated CONUT scores appear to align with characteristics often observed in frailty syndromes—such as reduced body mass, a greater burden of comorbidities, functional limitations, systemic inflammation, and anemia. This pattern may indicate that the CONUT score captures a broader, multidimensional risk profile encompassing nutritional status, inflammatory processes, and overall clinical severity. Similar trends have been noted in previous studies involving hospitalized older adults, where higher CONUT scores were linked to impaired body composition, inflammatory markers, and frailty, thereby supporting the construct validity of CONUT as a potential marker of vulnerability in geriatric populations [12].

The association observed between higher CONUT scores and male sex should be interpreted with caution. While some prior studies have reported a higher prevalence of malnutrition among men [24], others have not demonstrated consistent sex-based differences [7]. In this context, our results may reflect indirect associations rather than a direct sex effect. For instance, older men may be more likely to experience a higher comorbidity burden and increased exposure to nutritional risk factors, which could contribute to poorer nutritional status [25]. Taken together, these findings should be interpreted with caution, and further research may help to better understand sex-related nutritional differences in this population. The observed inverse relationship between CONUT scores and the KATZ Index may indicate that nutritional deficits are potentially linked to greater functional dependence.

Supporting this, Lo Buglio et al. (2024) found that older adults with high CONUT scores exhibited worse physical performance, greater muscle wasting, and lower Barthel Index scores [12]. These findings suggest a potential link between nutritional status and functional decline.

Another important clinical factor associated with higher CONUT scores in our cohort was the presence of malignancy. The association between high CONUT scores and malignancy may be explained by cancer-related cachexia, chronic inflammation, and metabolic disturbances. These conditions overlap with the components of the CONUT score, such as serum albumin, total lymphocyte count, and total cholesterol. Previous studies have demonstrated that the CONUT score may carry prognostic value in oncology populations, assisting in the prediction of clinical outcomes and informing supportive care strategies [26].

Higher CONUT scores were associated with lower BMI and higher comorbidity burden (CCI), suggesting that poor nutritional status may reflect both physical decline and systemic disease severity. While some studies have reported associations between the CONUT score, body composition, and frailty [12], a study by Rinninella et al. conducted in internal medicine and gastroenterology units did not find significant differences in BMI or CCI across CONUT score groups [7]. This discrepancy may be explained by differences in the clinical characteristics of the study populations. Further research is needed to better understand the association between CONUT scores, nutritional indicators, and comorbidities in older adults.

In our study, the observed association between higher CONUT scores and lower hemoglobin levels may be explained by known mechanisms through which malnutrition and chronic inflammation suppress erythropoiesis and disrupt iron metabolism [27].

Higher CONUT scores were also associated with elevated CRP levels, suggesting a link between systemic inflammation and poor nutritional status. This relationship may reflect the inflammatory cascade’s effects on appetite suppression, increased energy expenditure, and muscle catabolism—all of which contribute to nutritional decline [28]. Similarly, associations between elevated CONUT scores and higher CRP concentrations in elderly inpatients have been reported in the literature [12]. Taken together, these findings suggest that the CONUT score may reflect both nutritional status and inflammatory activity in clinically vulnerable populations.

### 4.3. Association of the CONUT Score with Ultrasound-Assessed Muscle Mass and Subcutaneous Fat Thickness

Beyond its association with clinical and biochemical parameters, the CONUT score has also been linked to alterations in body composition. Previous studies have demonstrated that higher CONUT scores are associated with reduced muscle mass, particularly among hospitalized older adults assessed using bioelectrical impedance analysis (BIA) [12]. In our study, a significant reduction in rectus femoris and biceps brachii muscle thickness, as assessed by ultrasonography, was observed in patients with high CONUT scores. This finding may reflect the combined adverse effects of protein-energy deficiency and systemic inflammation [12].

To better understand this relationship, the individual components of the CONUT score can be considered in relation to muscle health.

In this context, serum albumin, one of the core components of the CONUT score, is a classical biomarker that reflects protein reserves and overall nutritional status. Low serum albumin levels have been previously associated with reduced muscle mass and poor functional capacity [29]. Similarly, disturbances in lipid metabolism have been shown to negatively affect muscle health. Specifically, lower levels of total cholesterol and triglycerides have been linked to an increased risk of sarcopenia in older adults. These lipid abnormalities may impair muscle tissue through multiple mechanisms, including disrupted energy metabolism, altered muscle microenvironment, ectopic fat accumulation, and heightened inflammatory responses [30]. Another critical component of the CONUT score, lymphocyte count, also plays a key role in muscle maintenance. Lymphocytes—particularly CD8^+^ T cells and regulatory T cells—are known to contribute to muscle regeneration beyond their roles in immune surveillance. Through cytokine-mediated interactions with the muscle niche, they modulate muscle cell proliferation, migration, and differentiation. Thus, reductions in lymphocyte levels or function may contribute to muscle atrophy and the pathogenesis of sarcopenia [31].

Consistent with these associations, a study by Yamura et al. demonstrated that a CONUT score ≥ 4 was associated with reduced gait ability in older adults and identified it as an independent predictor of poor functional prognosis [32].

Moreover, the frequent coexistence of malnutrition and sarcopenia has been increasingly recognized in the literature, often referred to as the ‘malnutrition–sarcopenia syndrome’ [17]. Cereda and colleagues emphasized that nutritional evaluation in older adults should extend beyond conventional measures such as weight and BMI, incorporating additional parameters like muscle mass and systemic inflammation [33]. In line with this perspective, our observations may suggest that the CONUT score has the potential to reflect not only nutritional and inflammatory status, but also aspects of muscle mass and physical function. In this context, it may represent a promising candidate among next-generation screening tools [33].

Nonetheless, larger prospective, multicenter studies are warranted to validate the utility of the CONUT score as a comprehensive marker that integrates nutritional status, muscle health, and functional capacity.

In addition to having reduced muscle mass, patients with higher CONUT scores also exhibited lower subcutaneous fat thickness. In contrast, previous studies have reported a positive association between fat mass and CONUT scores in the context of body composition [12]. This discrepancy may be attributable to differences in clinical populations. In particular, patients receiving palliative care may experience concurrent losses in both fat and muscle mass due to factors such as inflammation, anorexia, and physical inactivity [34].

### 4.4. Prognostic Significance of Ultrasound-Assessed Muscle Mass

Relationship between muscle mass assessed by ultrasonographic methods and clinical outcomes has been frequently emphasized in the literature. For instance, in patients with head and neck cancer, a reduction in the cross-sectional area of the rectus femoris muscle over a 12-month follow-up period was found to be significantly associated with sarcopenia, malnutrition, and an increased risk of mortality [15]. Similarly, Veronese et al. demonstrated in their comprehensive review that sarcopenia is significantly associated with adverse outcomes such as mortality, disability, and falls [4].

However, in this study, although muscle mass was found to be significantly associated with the CONUT score, it did not demonstrate an independent prognostic value for 30-, 45-, or 60-day mortality. This apparent discordance may be explained by several factors. First, the patient population was clinically heterogeneous in terms of underlying diagnoses and disease severity.

Second, in palliative care settings, short-term mortality may be more strongly influenced by acute clinical deteriorations—such as infection, sepsis, or organ failure—rather than by chronic indicators like muscle mass or nutritional status [35].

Third, the relatively short follow-up period may have limited the ability to detect the long-term prognostic impact of structural muscle loss [5]. Structural measures, while in-formative about nutritional and functional reserves, might not adequately capture the immediate clinical risks faced by palliative patients. Taken together, these factors suggest that the absence of an independent prognostic role for muscle mass in our findings should be interpreted in light of the study’s methodological and clinical constraints.

In this context, further studies with longer observation periods and more homogeneous patient cohorts are warranted to better elucidate the prognostic significance of muscle mass in palliative care settings. Although muscle ultrasound was associated with the CONUT score, it did not emerge as an independent predictor of short-term mortality. Nevertheless, our findings may provide preliminary data to the literature, suggesting that muscle ultrasound has limited prognostic value in the context of geriatric palliative care and may serve as a complementary tool for assessing nutritional and functional status.

### 4.5. Prognostic Value of the CONUT Score and Malignancy

In our study, both a high CONUT score and the presence of malignancy were significantly associated with mortality across all time periods. The prognostic value of the CONUT score has been demonstrated in various studies [16,24,26]. This value may be related to its components—serum albumin, lymphocyte count, and total cholesterol—which reflect different pathophysiological dimensions.

Capurso et al. (2025) reported that low serum albumin levels in hospitalized older adults were associated with increased early mortality, likely due to underlying malnutrition, chronic inflammation, and diminished physiological reserves [36]. Moreover, malnutrition may increase the risk of mortality through several mechanisms, including frailty, delirium, immune dysfunction, muscle loss, and cognitive decline [37]. Low cholesterol levels, another component of the CONUT score, may reflect conditions such as malnutrition, frailty, and chronic inflammation in older adults, thereby contributing to an increased risk of mortality [38]. Similarly, lymphopenia has been identified as an independent risk factor for short-term in-hospital mortality in older adults. This may be explained by age-related decline in immune function, increased susceptibility to infections, and immunodeficiency [39].

In our study, the association between high CONUT scores and mortality may be explained by the fact that the components of the CONUT score (albumin, total cholesterol, and lymphocyte count) reflect key pathophysiological disturbances commonly observed in palliative care patients—such as malnutrition, inflammation, and immune dysfunction—which are known to be significant predictors of prognosis [2,10]. ROC analysis revealed a moderate predictive value, particularly at 60-day follow-up (AUC: 0.69–0.74). These findings suggest that the CONUT score may serve as a useful tool for identifying high-risk patients at the time of admission; however, its predictive value should be confirmed in larger, multicenter studies.

Furthermore, the independent association between the presence of malignancy and short-term (30-, 45-, and 60-day) mortality may be explained by the fact that palliative cancer patients are typically in advanced stages of disease, often with limited or no remaining treatment options. During this period, an increased tumor burden, along with acute complications such as infection, organ failure, or malignancy-related bleeding, may substantially heighten the risk of death within a short timeframe [40]. Nutritional status and the presence of malignancy may warrant more careful consideration during clinical decision-making in palliative care settings.

#### Limitations

This study has several limitations. It was conducted in a single tertiary palliative care center with a relatively small sample size, which may limit the generalizability of the findings. Muscle mass was assessed using ultrasound alone, without validation against gold-standard imaging techniques such as MRI, CT, or DXA. Nutritional status was evaluated solely with the CONUT score, and no comparison with other commonly used nutritional screening or assessment tools (e.g., MUST, NRS-2002, MNA) was made. As a result, it remains unclear whether the findings are specific to the CONUT score or reflect general malnutrition risk. This should be acknowledged as a methodological limitation of the study.

Additionally, the CONUT score was assessed at baseline only, and changes in nutritional status over time were not monitored. Cirrhosis and chronic kidney disease are known to affect serum albumin and total cholesterol levels independently of nutritional status. Therefore, patients with these conditions were excluded from our study. Although we did not encounter any cases of nephrotic syndrome during data collection, and it was not listed as an exclusion criterion, we acknowledge that it may also influence the CONUT score.

However, the exclusion of patients with chronic kidney disease, cirrhosis, or those receiving lipid-lowering therapy may limit the generalizability of our findings, given the high prevalence of these conditions and treatments in real-world palliative care populations.

In future studies, analyzing such comorbidities as a separate subgroup may help to better evaluate the validity and clinical relevance of the CONUT score.

Although high intra-rater reliability was confirmed in a healthy subgroup, this study did not assess inter-rater reliability or external reproducibility, which may limit the generalizability of the ultrasound findings. Future research involving multiple operators and external validation could help support the broader use of these measures in palliative care settings.

Owing to the relatively short follow-up and the lack of observed competing events, the application of alternative analytical approaches (e.g., a single primary endpoint, time-dependent Cox models, or competing-risk analyses) was limited in the present study. Future investigations with longer follow-up and more heterogeneous outcome events may further explore these methods.

While no significant statistical multicollinearity was observed among the variables included in the model, some of them—such as hemoglobin and the CONUT score—may reflect overlapping biological domains, particularly those related to nutritional and systemic health status. Due to the predefined analytical framework of the study, alternative modeling approaches excluding potentially overlapping variables were not applied. This should be acknowledged as a limitation when interpreting the independent prognostic value of the CONUT score. We believe that such sensitivity analyses would be valuable in future research, particularly in studies specifically designed to explore these methodological considerations.

Notably, the strengths of this study include its prospective design in a geriatric palliative care setting, the concurrent evaluation of nutritional status and muscle mass using the CONUT score and ultrasonography, and the analysis of the time-dependent prognostic value of the CONUT score at multiple follow-up points.

## 5. Conclusions

At the time of admission, higher CONUT scores were found to be associated with reduced muscle mass. Furthermore, a CONUT score ≥ 5 and the presence of malignancy independently predicted 30-, 45-, and 60-day mortality in geriatric palliative care patients. Given its simplicity and routine availability, the CONUT score may be considered a potentially useful bedside indicator for early risk stratification and care planning in this population. However, further studies are needed to confirm these findings and to compare its prognostic utility against other nutritional assessment tools.

## Figures and Tables

**Table 1:** Screening tool for Controlling Nutritional Status.

Serum Albumin (g/dL)		Total Cholesterol (mg/dL)		Lymphocytes (/mm^3^)	
	score		score		score
3.5–4.5	0	>180	0	>1600	0
3.0–3.49	2	140–180	1	1200–1599	1
2.5–2.9	4	100–139	2	800–1199	2
<2.5	6	<100	3	<800	3

Nutritional status: normal 0–1; mild 2–4; moderate 5–8; severe 9–12.

**Table 2:** Baseline characteristics and comparisons according to CONUT risk categories.

			CONUT Score		
		Total (*n* = 200)	≤4 (*n* = 62)	≥5 (*n* = 138)	*p*
Age, years *	78.4 ± 10.2		77.6 ± 10.4	78.3 ± 10.3	0.496
Sex (*n*, %)	female	109 (54.5%)	41 (37.6%)	68 (62.4%)	0.027
	male	91 (45.5%)	21 (23.1%)	70 (76.9%)	
Nutritional route (*n*, %)	oral	112 (56%)	41 (36.6%)	71 (63.4%)	*0.053*
	non-oral	88 (44%)	21 (23.9%)	67 (76.1%)	
KATZ (*n*, %)	independent	12 (6%)	10 (83.3%)	2 (16.7%)	<0.001
	dependent	188 (94%)	52(27.7%)	136 (72.3%)	
HT (*n*, %)	no	94 (47%)	23 (24.5%)	71 (75.5%)	0.060
	yes	106 (53%)	39 (36.8%)	67 (63.2%)	
DM (*n*, %)	no	137 (68.5%)	44 (32.1%)	93 (67.9%)	0.615
	yes	63 (31.5%)	18 (28.6%)	45 (71.4%)	
Cancer (*n*, %)	no	139 (69.5%)	54(38.8%)	85 (61.2%)	<0.001
	yes	61 (30.5%)	8(13.1%)	53 (86.9%)	
Stroke (*n*, %)	no	147 (73.5%)	51 (34.7%)	96 (65.3%)	0.060
	yes	53 (26.5%)	11 (20.8%)	42 (79.2%)	
Dementia (*n*, %)	no	130 (65%)	36 (27.7%)	94 (72.3%)	0.168
	yes	75 (35%)	26 (37.1%)	44 (62.9%)	
30-day Survival (*n*, %)	survived	147 (73.5%)	56 (38.1%)	91 (61.9%)	<0.001
	deceased	53 (26.5%)	6 (11.3%)	47 (88.7%)	
45-day Survival (*n*, %)	survived	135 (67.5%)	55 (40.7%)	80 (59.3%)	<0.001
	deceased	65 (32.5%)	7 (10.8%)	58 (89.2%)	
60-day Survival (*n*, %)	survived	126 (63.0%)	55 (43.7%)	71 (56.3%)	<0.001
	deceased	74 (37.0%)	7 (9.5%)	67 (90.5%)	
Survival Time (days) **		28.5 (14–54.7)	29 (14–53.5)	28.5 (14–55)	0.873
BMI, kg/m^2^ **		22.4 (20–25.9)	23.4 (21–27)	22 (19.5–25.4)	0.050
CCI score **		7 (5–8)	6 (4–7)	7 (6–8.2)	<0.001
Albumin (g/dL) **		2.8 (2.5–3.3)	3.4 (3.1–3.8)	2.6 (2.3–2.9)	<0.001
Total cholesterol (mg/dL) **		138 (114–166)	158 (137–191)	128 (109–153)	<0.001
Lymphocytes (10^9^/L) **		1525 (1045–2200)	2000 (1612–2512)	1285 (947–1865)	<0.001
CONUT score **		6 (4–8)	3 (1–4)	7 (6–9)	<0.001
Hb (g/dL) **		9.9 (8.6–11.1)	11 (10–12.2)	9.3 (8.3–10.7)	<0.001
Creatinine (mg/dL) **		0.7 (0.5–1)	0.74 (0.5–1)	0.64 (0.4–1)	0.731
CRP **		39 (14–88)	11.5 (3–41)	51(26–102)	<0.001
RF-SFT (cm) **		0.65 (0.42–0.97)	0.75 (0.5–1)	0.6 (0.4–0.9)	0.008
RF-MT (cm) **		0.58 (0.46–0.69)	0.64 (0.5–0.7)	0.5 (0.4–0.6)	<0.001
RF-CSA (cm^2^) **		1.58 (1.23–1.95)	1.76 (1.5–2.1)	1.4 (1.1–1.8)	<0.001
BB-SFT (cm) **		0.3 (0.21–0.41)	0.29 (0.2–0.4)	0.31 (0.2–0.4)	0.819
BB-MT (cm) **		0.93 (0.8–1.1)	1.04 (0.8–1.1)	0.92(0.8–1)	0.006
BB-CSA (cm^2^) **		2.64 (2.2–3.2)	2.94 (2.3–3.5)	2.6 (2.1–3)	0.013

Abbreviations: BB-CSA = biceps brachii cross-sectional area; BMI = body mass index; BB-MT = biceps brachii muscle thickness; BB-SFT = biceps brachii subcutaneous fat thickness; CCI = Charlson Comorbidity Index; CONUT = Controlling Nutritional Status; DM = diabetes mellitus; HT = hypertension; Non-oral = enteral and/or parenteral; RF-CSA = rectus femoris cross-sectional area; RF-MT = rectus femoris muscle thickness; RF-SFT = rectus femoris subcutaneous fat thickness; SD = standard deviation; * = mean ± standard deviation; ** = median/interquartile ranges (p25–p75).

**Table 3:** Correlation Between CONUT Score and Muscle Parameters.

Muscle Parameter	Spearman’s Rho (r)	*p*
RF-SFT (cm)	−0.210	0.003
RF-MT (cm)	−0.271	0.001
RF-CSA (cm^2^)	−0.308	<0.001
BB-SFT (cm)	−0.04	0.570
BB-MT (cm)	−0.261	<0.001
BB-CSA (cm^2^)	−0.240	0.001

Abbreviations: BB-CSA = biceps brachii cross-sectional area; BB-MT = biceps brachii muscle thickness; BB-SFT = biceps brachii subcutaneous fat thickness; RF-CSA = rectus femoris cross-sectional area; RF-MT = rectus femoris muscle thickness; RF-SFT = rectus femoris subcutaneous fat thickness.

**Table 4:** Cox regression analysis of factors associated with 30-day, 45-day, and 60-day mortality.

	Univariate					*p*	Multivariable					*p*
	β	HR	95% CI				β	HR	95% CI			
30 day Mortality												
Age	−0.011	0.989	0.964	-	1.015	0.422						
Sex	0.444	1.559	0.905		2.683	0.109						
BMI	−0.032	0.968	0.914		1.026	0.275						
Hb	0.008	1.008	0.865		1.175	0.917						
CCI score	0.161	1.175	1.039	-	1.328	0.010 *						
Cancer	1.247	3.479	2.014	-	6.009	<0.001 *	1.107	3.025	1.741	-	5.256	<0.001 *
CONUT score ≥ 5	1.276	3.582	1.531	-	8.379	<0.003 *	1.051	2.862	1.212	-	6.755	0.016 *
RF-SFT (cm)	−0.292	0.747	0.364		1.533	0.426						
RF-MT (cm)	−0.178	0.837	0.190	-	3.687	0.055						
BB-MT (cm)	−1.031	0.357	0.102	-	1.241	0.105						
45 day Mortality												
Age	−0.018	0.983	0.960	-	1.006	0.136						
Sex	0.383	1.466	0.899		2.391	0.125						
BMI	−0.029	0.972	0.923		1.023	0.274						
Hb	−0.028	0.972	0.847		1.117	0.691						
CCI score	0.186	1.204	1.075	-	1.348	0.001 *						
Cancer	1.274	3.577	2.183	-	5.860	<0.001 *	1.142	3.132	1.903	-	5.155	0.006 *
CONUT score ≥ 5	1.324	3.759	1.716	-	8.237	0.001 *	1.113	3.043	1.380	-	6.711	<0.001 *
RF-SFT (cm)	−0.489	0.613	0.312		1.204	0.156						
RF-MT (cm)	−0.192	0.825	0.215	-	3.172	0.780						
BB-MT (cm)	−1.076	0.341	0.109	-	1.070	0.065						
60 day Mortality												
Age	−0.013	0.987	0.966	-	1.008	0.235						
Sex	0.376	1.456	0.920		2.303	0.108						
BMI	−0.027	0.974	0.928		1.021	0.273						
Hb	−0.060	0.942	0.827		1.072	0.364						
CCI score	0.170	1.185	1.064	-	1.321	0.002 *						
Cancer	1.162	3.196	2.018	-	5.060	<0.001 *	1.039	2.826	1.777	-	4.494	<0.001 *
CONUT score ≥ 5	1.471	4.355	1.999	-	9.487	<0.001 *	1.314	3.723	1.702	-	8.143	<0.001 *
RF-SFT (cm)	−0.403	0.668	0.356		1.254	0.209						
RF-MT (cm)	−0.182	0.834	0.232	-	2.994	0.781						
BB-MT (cm)	−0.904	0.405	0.141	-	1.161	0.092						

Abbreviations: BMI = body mass index; BB-MT = biceps brachii muscle thickness; CCI = Charlson Comorbidity Index; CONUT = Controlling Nutritional Status; Hb = hemoglobin; RF-MT = rectus femoris muscle thickness; RF-SFT = rectus femoris subcutaneous fat thickness; HR = hazard ratio; CI = confidence interval. Dependent variable: 30-, 45-, and 60-day mortality. Independent variables: Age, sex, BMI, CCI score, cancer status, CONUT score ≥ 5, Hb, RF-SFT, RF-MT, and BB-MT. *p* < 0.05 was considered statistically significant. * Indicates statistically significant results.

**Table 5:** Diagnostic Accuracy of the CONUT Score in Predicting Short-Term Mortality.

Mortality Day	AUC	Optimal Cut-off	Sensitivity (%)	Specificity (%)	Youden Index	PPV (%)	NPV (%)
30-day	0.694	6.50	58.5	70.1	0.286	41.3	82.4
45-day	0.706	4.50	89.2	40.7	0.299	42.0	88.7
60-day	0.736	4.50	90.5	43.7	0.342	48.6	88.7

Abbreviations: AUC = area under the receiver operating characteristic curve; PPV = positive predictive value; NPV = negative predictive value; Youden Index = sensitivity + specificity − 1; Sensitivity = true positive rate; Specificity = true negative rate.; Optimal Cut-off = CONUT score threshold maximizing Youden Index.

## Data Availability

The data presented in this study are available from the author upon reasonable request.

## Abbreviations

ADL	Activities of Daily Living
BB	Biceps brachii
BB-CSA	Biceps brachii cross-sectional area
BB-MT	Biceps brachii muscle thickness
BB-MT/SFT	Biceps muscle thickness to subcutaneous fat thickness ratio
BB-SFT	Biceps brachii subcutaneous fat thickness
BMI	Body mass index
CCI	Charlson Comorbidity Index
CONUT	Controlling Nutritional Status
CRP	C-reactive protein
CSA	Cross-sectional area
CT	Computed tomography
DM	Diabetes Mellitus
DXA	Dual-energy X-ray absorptiometry
ESPEN	European Society for Clinical Nutrition and Metabolism
EWGSOP2	European Working Group on Sarcopenia in Older People 2
GNRI	Geriatric Nutritional Risk Index
HT	Hypertension
ICC	Intraclass correlation coefficient
MNA-SF	Mini Nutritional Assessment–Short Form
MRI	Magnetic resonance imaging
MUST	Malnutrition Universal Screening Tool
NRS-2002	Nutritional Risk Screening 2002
RF	Rectus femoris
RF-CSA	Rectus femoris cross-sectional area
RF-MT	Rectus femoris muscle thickness
RF-MT/SFT	Rectus femoris muscle thickness to subcutaneous fat thickness ratio
RF-SFT	Rectus femoris subcutaneous fat thickness
SD	Standard deviation
SFT	Subcutaneous fat thickness
SPSS	Statistical package for social sciences
USG	Ultrasonography
